# Impact of nonsynonymous single nucleotide polymorphisms in *PROCR* gene on protein stability and thrombotic risk: a molecular dynamic approach

**DOI:** 10.3389/fgene.2025.1580993

**Published:** 2025-04-30

**Authors:** Hytham Ahmed Abuagla, Khalid Mohamed Adam, Mohamed E. Elangeeb, Elsadig Mohamed Ahmed, Elshazali W. Ali, Ali M. Edris, Abubakr Ali Elamin MohamedAhmed, Elmoiz Idris Eltieb, Tarig Babikir Algak Khalid, Bahaeldin K. Elamin, Hiba Mahgoub Ali Osman, Ebtehal Salih Idris

**Affiliations:** ^1^ Department of Medical Laboratory Sciences, College of Applied Medical Sciences, University of Bisha, Bisha, Saudi Arabia; ^2^ Department of Pathology, College of Medicine, University of Bisha, Bisha, Saudi Arabia; ^3^ Department of Microbiology and Clinical Parasitology, College of Medicine, University of Bisha, Bisha, Saudi Arabia

**Keywords:** thrombosis, PROCR, nsSNPs, EPCR, protein stability, molecular dynamics

## Abstract

**Introduction:**

Thrombosis is a serious vascular disorder influenced by genetic factors, including nonsynonymous single nucleotide polymorphisms (nsSNPs) in the *PROCR* gene, which encodes the endothelial protein C receptor (EPCR). These mutations may disrupt EPCR stability and impair its anticoagulant function, thereby increasing the risk of thrombosis.

**Methods:**

We employed a multi-layered computational approach to analyze 217 nsSNPs in the *PROCR* gene. Functional impacts were predicted using Sorting Intolerant From Tolerant (SIFT), Polymorphism Phenotyping v2 (PolyPhen-2), Screening for Non-Acceptable Polymorphisms 2 (SNAP2), and Protein Analysis Through Evolutionary Relationships (PANTHER). Disease associations were assessed using Single Nucleotide Polymorphisms and Gene Ontology (SNP&GO) and Predictor of Human Deleterious Single Nucleotide Polymorphisms (PhD-SNP). Protein stability was evaluated using I-Mutant and MUpro, while structural implications were analyzed with Mutation Prediction (MutPred), ConSurf, and Have Our Protein Explained (HOPE). Active binding sites were identified using PyMOL. Finally, 100-nanosecond molecular dynamics (MD) simulations were conducted using GROningen MAchine for Chemical Simulations (GROMACS) to compare structural deviations, flexibility, and solvent interactions between wild-type EPCR and key mutant proteins.

**Results:**

Our integrated analysis identified three high-risk nsSNPs—T174I, N136I, and L168P—that detrimentally affect EPCR function. These variants disrupt critical glycosylation sites, α-helix integrity, and catalytic residues, leading to increased root mean square deviation (RMSD) and root mean square fluctuation (RMSF), reduced hydrogen bonding, and higher solvent-accessible surface area (SASA) in mutants compared to the wild-type. Disease association tools further linked these mutations to an elevated thrombotic risk.

**Discussion:**

These findings suggest that the identified nsSNPs destabilize EPCR by altering its structural dynamics and reducing its capacity to activate protein C. This provides mechanistic insight into how *PROCR* variation may contribute to thrombotic disorders and highlights the utility of *in silico* approaches for prioritizing potentially pathogenic variants.

**Conclusion:**

Our study demonstrates that deleterious nsSNPs in the *PROCR* gene can significantly impair EPCR stability and function, thereby increasing susceptibility to thrombosis. These findings provide a foundation for future experimental validation and may inform the development of personalized therapeutic strategies for managing thrombotic disorders.

## 1 Introduction

Thrombosis is a severe health disorder characterized by the formation of blood clots that restrict or block normal blood flow through the circulatory system (Koupenova et al., 2017). This can result in serious conditions such as stroke, myocardial infarction (Mackman, 2012), or venous thromboembolism. The endothelial protein C receptor (EPCR), encoded by the *PROCR* gene, is a key component of the anticoagulation pathway. It facilitates the activation of protein C, which plays a critical role in degrading the procoagulant factors Va and VIIIa and directly inhibiting the formation of pathological clots (Dahlbäck and Villoutreix, 2005). Genetic variants, particularly nonsynonymous single nucleotide polymorphisms (nsSNPs), in the *PROCR* gene can significantly alter the structure and function of the endothelial protein C receptor (EPCR). EPCR plays a crucial anticoagulant role by binding to activated protein C (APC) and enhancing its proteolytic inactivation of coagulation factors Va and VIIIa, thereby reducing thrombin generation. Mutations that destabilize EPCR or disrupt its APC-binding region can impair this anticoagulant function. Some nsSNPs lead to reduced cell-surface expression of EPCR or modify its glycosylation pattern, affecting receptor trafficking and recycling. Others enhance the shedding of soluble EPCR (sEPCR), which competes with membrane-bound EPCR for APC binding and promotes a prothrombotic state. For instance, the Ser219Gly variant has been associated with elevated sEPCR levels and an increased risk of venous thromboembolism and myocardial infarction. While nsSNPs may also influence gene expression, splicing, or protein trafficking, this study focuses specifically on their effects on EPCR’s structural stability and conformation (Reiner et al., 2008; Kallel et al., 2012). This emphasis is grounded in the fact that EPCR function depends critically on the integrity of its three-dimensional structure, particularly the ligand-binding groove and membrane-anchoring domains. Alterations in folding or flexibility can disrupt APC interaction, increase susceptibility to proteolytic cleavage, or reduce cell-surface localization—mechanisms that directly elevate thrombotic risk. Furthermore, many deleterious nsSNPs exert their effects at the post-translational level rather than at the transcriptional level. Thus, structural investigation through molecular dynamics simulations offers a mechanistic framework for understanding how specific amino acid substitutions may destabilize EPCR and compromise its anticoagulant role (Saposnik et al., 2004; Qu et al., 2007; Medina et al., 2005). Previous studies have emphasized the gene’s importance in maintaining vascular integrity and proper blood coagulation (Esmon, 2003; Van de Wouwer et al., 2004; Feistritzer and Riewald, 2005; Taylor et al., 2001). Specifically, nsSNPs that induce amino acid substitutions are known to affect both the structural and functional integrity of the EPCR protein (Van de Wouwer et al., 2004; Feistritzer and Riewald, 2005; Fukudome and Esmon, 1994). These genetic variants may lead to either gain or loss of function, both of which are associated with an increased risk of thrombosis (Taylor et al., 2001). To date, no comprehensive mechanistic studies have elucidated how these nsSNPs affect EPCR stability and function. Molecular dynamics simulations have increasingly been employed to explore the impact of genetic variation on protein structure and function (Hollingsworth and Dror, 2018; Lemkul, 2018; Shaw et al., 2010; Karplus and McCammon, 2002). These simulations allow researchers to track protein behavior in a near-native environment over time, offering insights into the stability of both wild-type and mutant proteins.

This gene has been reported to be crucial for maintaining vascular integrity and blood coagulation by former studies (Esmon, 2003; Van de Wouwer et al., 2004; Feistritzer and Riewald, 2005; Taylor et al., 2001). Mutations in the *PROCR* gene are known to affect both the structural and functional integrity of EPCR protein, especially nsSNPs that induce amino acid substitutions (Van de Wouwer et al., 2004; Feistritzer and Riewald, 2005; Fukudome and Esmon, 1994). These mutations can be gain or loss of function, and both result in an increased thrombotic risk (Taylor et al., 2001). No detailed mechanistic study on how these nsSNPs specifically affect the stability and function of EPCR protein could be identified. Molecular dynamics simulations have been increasingly used to explore the consequences of genetic variation disrupting protein structure and function (Hollingsworth and Dror, 2018; Lemkul, 2018; Shaw et al., 2010; Karplus and McCammon, 2002). Using these simulations, thereby following the behavior of proteins in their natural environment over time gives us access to not only wild-type protein stability dynamics but also dynamics associated with disease mutations. This study aims to identify and characterize the functional impact of deleterious nonsynonymous single nucleotide polymorphisms (nsSNPs) in the *PROCR* gene using an integrative computational approach. Specifically, we seek to determine how these mutations may affect protein structure, stability, and function, and whether they may contribute to thrombotic risk. We hypothesize that certain nsSNPs, particularly those occurring in conserved or functionally important domains of EPCR, induce conformational changes that impair ligand binding, alter structural stability, or increase solvent accessibility. To test this hypothesis, we employed a multi-step workflow involving variant prioritization, conservation analysis, structural modeling, and molecular dynamics simulations.

## 2 Materials and methods

### 2.1 Work plan

**FIGURE 1:**
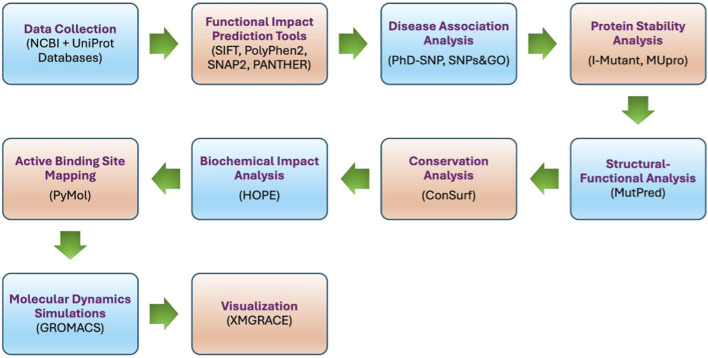
Work plan flow.

The work plan (Figure 1) involves analyzing missense SNPs in the *PROCR* gene using data from the SNP database (dbSNP) of the National Center for Biotechnology Information (NCBI) and Universal Protein Resource (UniProt). Functional impacts were predicted with SIFT, PolyPhen2, SNAP2, and PANTHER, while disease associations with thrombosis were assessed using PhD-SNP and SNPs&GO. Protein stability effects were quantified with I-Mutant and MUpro, and structural-functional implications were evaluated using MutPred. Conservation analysis was performed with ConSurf, biochemical impacts with HOPE, and active binding sites were mapped using PyMol. Molecular dynamics simulations in GROMACS provided structural insights analyzed with tools like g_rms and g_rmsf and visualized in XMGRACE.

### 2.2 Data collection

Missense SNP data for the *PROCR* gene were collected from the dbSNP database (NCBI) (https://www.ncbi.nlm.nih.gov) using gene ID 10544 and accession number NG_032899.2. In addition, the UniProt ID Q9UNN8 and PDB ID: 4V3D, were used to retrieve EPCR protein sequences and functional annotations from the UniProt database (https://www.uniprot.org) for further analysis.

### 2.3 SNPs-based functional impact scoring

The effect of the identified SNPs on EPCR protein function was evaluated using several predictive bioinformatics tools to provide solid and reliable predictions. SIFT, the application was used to determine whether an amino acid substitution affects the function of a protein by exploiting sequence homology and physical characteristics of the amino acids in the proteins (https://sift.bii.a-star.edu.sg/www/SIFT_seq_submit2.html) (Choi and Chan, 2015). PolyPhen2 was used to predict the possible impact of an amino acid substitution on the structure and function of a protein, considering sequence and structure-based features (http://genetics.bwh.harvard.edu/pph2/) (Adzhubei et al., 2013). SNAP2 is a method to predict the functional effect of SNPs by using neural networks trained on a data set of experimental mutation data (https://github.com/Rostlab/SNAP2) (Hecht et al., 2015). PANTHER version 14.0 was used to predict the impact of amino acid substitutions on protein function based on evolutionary relationships among proteins (https://www.pantherdb.org/tools/csnpScoreForm.jsp) (Mi et al., 2019).

### 2.4 Identification of disease associations of SNPs

The following predictive tools were utilized to forecast if a given SNP was likely associated with disease, especially thrombosis. PhD-SNP was used to predict disease-associated variants according to protein sequences (https://snps.biofold.org/phd-snp/phd-snp.html) (Capriotti and Fariselli, 2023). SNPS&GO, a genome-wide resource for functional-disease associations using region-based coding and non-coding variant-to-gene mappings (https://snps-and-go.biocomp.unibo.it/snps-and-go/) (Calabrese et al., 2009).

### 2.5 Quantifying the effect of SNPs on protein stability

The following predictive tools were used to assess the effect of SNPs on EPCR protein stability. I-Mutant predicted changes in protein stability caused by SNPs using both the sequence and structure of the protein (https://folding.biofold.org/i-mutant/i-mutant2.0.html) (Capriotti et al., 2005). MUpro, used support vector machines and neural networks to determine the effect of SNPs on protein stability, a source of extra predictive power (https://mupro.proteomics.ics.uci.edu) (Cheng et al., 2006).

### 2.6 Predicting structural and functional consequences

The MutPred Server was used to predict the potential impact of SNPs on the structure and function of EPCR protein. This system also incorporates several algorithms that predict structural and functional consequences of amino acid substitutions (http://mutpred.mutdb.org/#qform) (Pejaver et al., 2020).

### 2.7 Analysis of protein sequence conservation using the ConSurf server

Evolutionary conservation of amino acid residues within the EPCR protein was analyzed using ConSurf tool at an online server (http://consurf.tau.ac.il/). This evaluation provided information about which residues are likely functionally important based on conservation scores for homologous sequences (Ashkenazy et al., 2016).

### 2.8 Proteins: biochemical and structural analysis

Using the HOPE tool, SNPs in EPCR protein were analyzed for their biochemical properties and structural implications. We fed these mutant sequences for the predictive algorithm of HOPE and took insight into the vessel layer they may affect, including the effect on the function and 3D structure of the protein (https://www3.cmbi.umcn.nl/hope/input/) (Venselaar et al., 2010).

### 2.9 Identifying protein active sites

Structural visualization and mapping of putative active binding sites on EPCR protein were done using PyMol software (Version 2.5.4). This was indicated as an important survey for searching out that SNPs might affect protein binding with other molecules and give a clue for supporting functional disruption (Faure et al., 2019). In addition to PyMOL-based structural visualization, the CASTp server was also used. CASTp predicted a large pocket near residues N136, L168, and T174, which were subsequently prioritized for molecular dynamics simulations.

### 2.10 Molecular dynamics simulations (MDS)

Molecular dynamics simulations were performed using GROMACS 2021.3 (Lindahl et al., 2023) to investigate structural and dynamic differences between wild-type EPCR and three mutants; L168P, N136I, and T174I. The OPLS-AA force field was applied to parameterize the systems. Each protein was solvated in a cubic water box with a 1.0 nm buffer distance, and charge neutrality was achieved by adding ten sodium (Na^+^) ions using the *genion* tool. Energy minimization was conducted via the steepest descent algorithm (50,000 steps) to eliminate steric clashes. Equilibration followed under NVT (constant particle number, volume, temperature) and NPT (constant particle number, pressure, temperature) ensembles for 100 ns, with temperature maintained at 310 K using the Berendsen thermostat and pressure at 1 bar using the Parrinello-Rahman barostat. Production runs were extended to 100 ns for both wild-type and mutant systems, with trajectory data saved at 1 ns intervals to capture structural evolution (Abraham et al., 2015).

Structural metrics were computed using GROMACS modules RMSD, RMSF, Radius of gyration (Rg), Hydrogen bonds, and Solvent-accessible surface area SASA. Trajectories were visualized and plotted using XMGRACE to compare wild-type and mutant systems (Van Der Spoel et al., 2005).

To validate the reliability of our computational workflow, we retrospectively, included the well-characterized S219G (rs867186) mutation in the initial screening. This variant has been previously associated with increased soluble EPCR levels and elevated thrombotic risk in several GWAS and clinical studies. In our analysis, this mutation was predicted as deleterious by MuPro with prediction that it decreases the protein stability, PhD-SNP, and HOPE suggesting a possible effect on the contact with the lipid membrane. Its concordance with known clinical data supports the predictive accuracy of our computational pipeline. Although S219G was not selected for full MD simulation due to its distal location from the reactive surface, its inclusion in the screening phase provided internal validation. This reinforces the rationale for selecting other novel nsSNPs (e.g., N136I, L168P, T174I) that exhibit similar or greater predicted destabilizing potential.

## 3 Results

We collected data for 217 nsSNPs in the *PROCR* gene from the NCBI database to predict their effect on protein function.

### 3.1 Functional predictions and disease associations

Table 1 summarizes the functional predictions for a series of *PROCR* gene nsSNPs as determined by SIFT, PANTHER, PolyPhen-2, and SNAP2. All analyzed variants, including T174I, F36C, T233I, Y89H, and others, were consistently predicted to affect protein function. SIFT scores were uniformly low (ranging from 0.00 to 0.03), while both PANTHER and PolyPhen-2 predominantly classified these substitutions as possibly or probably damaging. SNAP2 further reinforced these predictions by indicating a detrimental effect. These convergent computational results strongly suggest that the identified nsSNPs compromise the normal function of the *PROCR* protein.

**TABLE 1:** Functional Predictions of *PROCR* gene nsSNPs.

SNPs information			SIFT		Panther		Polyphen2		SNAP2		
SNP-ID	Nucleotide Substitution	Amino Acid Substitution	Prediction	Score	Message	Pdel	Prediction	Score	Prediction	Score	Accuracy %
rs148819393	C>T	T174I	Affect Protein Function	0.00	Possibly damaging	0.5	Probably damaging	1	effect	43	0.71
rs199906882	T>G	F36C	Affect Protein Function	0.00	Possibly damaging	0.5	Probably damaging	1	effect	59	0.75
rs372109719	C>T	T233I	Affect Protein Function	0.00	Possibly damaging	0.5	Probably damaging	0.972	effect	53	0.75
rs372548432	T>C	Y89H	Affect Protein Function	0.00	Possibly damaging	0.5	Probably damaging	1	effect	59	0.75
rs745926875	T>G	I229S	Affect Protein Function	0.00	Possibly damaging	0.5	Probably damaging	1	effect	64	0.80
rs746421195	T>C	L165P	Affect Protein Function	0.01	Possibly damaging	0.5	Probably damaging	1	effect	18	0.59
rs746777605	C>T	T55M	Affect Protein Function	0.00	Possibly damaging	0.5	Probably damaging	1	effect	48	0.71
rs751051139	C>A	T185N	Affect Protein Function	0.00	Possibly damaging	0.5	Probably damaging	1	effect	49	0.71
rs755862059	C>T	P109L	Affect Protein Function	0.00	Possibly damaging	0.5	Probably damaging	1	effect	62	0.80
rs758409921	T>G	W79G	Affect Protein Function	0.00	Possibly damaging	0.5	Probably damaging	1	effect	65	0.80
rs761318857	G>C	R206P	Affect Protein Function	0.00	Possibly damaging	0.5	Probably damaging	0.999	effect	59	0.75
rs762010333	A>T	N136I	Affect Protein Function	0.02	Possibly damaging	0.5	Probably damaging	1	effect	70	0.80
rs766316108	G>T	R175L	Affect Protein Function	0.00	Possibly damaging	0.5	Probably damaging	1	effect	54	0.75
rs780324680	T>C	L168P	Affect Protein Function	0.00	Possibly damaging	0.5	Probably damaging	1	effect	70	0.80
rs866390428	T>C	L86P	Affect Protein Function	0.01	Possibly damaging	0.5	Probably damaging	1	effect	68	0.80
rs1209770143	G>A	G218D	Affect Protein Function	0.00	Possibly damaging	0.5	Probably damaging	1	effect	83	0.91
rs1275956595	A>G	Y89C	Affect Protein Function	0.00	Possibly damaging	0.5	Probably damaging	1	effect	46	0.71
rs1287887738	C>T	P109S	Affect Protein Function	0.00	Possibly damaging	0.5	Probably damaging	1	effect	50	0.75
rs1317140168	C>T	R104W	Affect Protein Function	0.03	Possibly damaging	0.5	Probably damaging	1	effect	75	0.80
rs1388488537	T>C	C232R	Affect Protein Function	0.00	Possibly damaging	0.5	Probably damaging	1	effect	81	0.91
rs1388649131	A>C	T233P	Affect Protein Function	0.00	Possibly damaging	0.5	Probably damaging	0.993	effect	72	0.80
rs1568591387	C>T	R175W	Affect Protein Function	0.00	Possibly damaging	0.5	Probably damaging	1	effect	65	0.80
rs2085990908	C>A	T66K	Affect Protein Function	0.02	Possibly damaging	0.5	Probably damaging	0.993	effect	62	0.80
rs2086024485	G>A	G235R	Affect Protein Function	0.00	Possibly damaging	0.5	Possibly damaging	0.797	effect	75	0.80

The disease association analysis performed using SNP&GO and PhD-SNP as shown in (Table 2), indicates that several of these nsSNPs are linked to disease. Variants such as T174I, F36C, I229S, and L165P, among others, are associated with disease at varying reliability indices. This robust prediction of disease involvement supports the clinical relevance of these mutations, potentially implicating them in increased thrombotic risk.

**TABLE 2:** Disease Associations of *PROCR* gene nsSNPs.

SNPs information			SNP&GO		PhD	
SNP-ID	Nucleotide Substitution	Amino Acid Substitution	Result	Reliability Index	Result	Reliability Index
rs148819393	C>T	T174I	Disease	3	Disease	5
rs199906882	T>G	F36C	Disease	7	Disease	7
rs745926875	T>G	I229S	Disease	8	Disease	5
rs746421195	T>C	L165P	Disease	7	Disease	5
rs751051139	C>A	T185N	Disease	4	Disease	3
rs755862059	C>T	P109L	Disease	7	Disease	4
rs758409921	T>G	W79G	Disease	5	Disease	1
rs761318857	G>C	R206P	Disease	6	Disease	4
rs762010333	A>T	N136I	Disease	7	Disease	6
rs780324680	T>C	L168P	Disease	8	Disease	6
rs866390428	T>C	L86P	Disease	7	Disease	3
rs1209770143	G>A	G218D	Disease	6	Disease	7
rs1275956595	A>G	Y89C	Disease	7	Disease	4
rs1317140168	C>T	R104W	Disease	7	Disease	6
rs1388488537	T>C	C232R	Disease	7	Disease	4
rs1568591387	C>T	R175W	Disease	6	Disease	1
rs2086024485	G>A	G235R	Disease	3	Disease	4

### 3.2 Impact on protein stability and function

The impact of the nsSNPs on protein stability as predicted by I-Mutant and MuPro shown in (Table 3). With the exception of the N136I variant, which shows a discrepancy between the two tools (I-Mutant predicts decreased stability whereas MuPro suggests a slight increase), the majority of mutations are predicted to decrease protein stability. These findings imply that the structural integrity of the *PROCR* protein is likely compromised by most of these variants, which may adversely affect its biological function (Table 4). details the predicted functional consequences using MutPred. For instance, the T174I variant (score 0.538) is predicted to cause the loss of N-linked glycosylation at N172, while F36C (0.754) is associated with the loss of sulfation at Y35. Other variants, including R206P, N136I, L168P, and G235R, are predicted to disrupt critical structural features or post-translational modifications, such as the loss of pyrrolidone carboxylic acid at Q203, further loss of N-linked glycosylation, and even the loss of a catalytic site, thereby potentially impairing the protein’s regulatory functions.

**TABLE 3:** Impact of nsSNPs in *PROCR* gene on protein Stability.

SNPs information			I-mutant		MuPro
SNP-ID	Nucleotide Substitution	Amino Acid Substitution	Result	Reliability Index	Result
rs148819393	C>T	T174I	Decrease protein stability	6	Decrease protein stability
rs199906882	T>G	F36C	Decrease protein stability	6	Decrease protein stability
rs745926875	T>G	I229S	Decrease protein stability	8	Decrease protein stability
rs746421195	T>C	L165P	Decrease protein stability	5	Decrease protein stability
rs751051139	C>A	T185N	Decrease protein stability	2	Decrease protein stability
rs755862059	C>T	P109L	Decrease protein stability	6	Decrease protein stability
rs758409921	T>G	W79G	Decrease protein stability	8	Decrease protein stability
rs761318857	G>C	R206P	Decrease protein stability	0	Decrease protein stability
rs762010333	A>T	N136I	Decrease protein stability	1	Increase protein stability
rs780324680	T>C	L168P	Decrease protein stability	6	Decrease protein stability
rs866390428	T>C	L86P	Decrease protein stability	2	Decrease protein stability
rs1209770143	G>A	G218D	Decrease protein stability	2	Decrease protein stability
rs1275956595	A>G	Y89C	Decrease protein stability	0	Decrease protein stability
rs1317140168	C>T	R104W	Decrease protein stability	5	Decrease protein stability
rs1388488537	T>C	C232R	Decrease protein stability	4	Decrease protein stability
rs1568591387	C>T	R175W	Decrease protein stability	4	Decrease protein stability
rs2086024485	G>A	G235R	Decrease protein stability	6	Decrease protein stability

**TABLE 4:** Impact of *PROCR* gene nsSNPs in Protein Function.

SNPs information			MutPred	
SNP-ID	Nucleotide Substitution	Amino Acid Substitution	Score	Function Affected
rs148819393	C>T	T174I	0.538	Loss of N-linked glycosylation at N172
rs199906882	T>G	F36C	0.754	Loss of Sulfation at Y35
rs761318857	G>C	R206P	0.546	Loss of Pyrrolidone carboxylic acid at Q203
rs762010333	A>T	N136I	0.775	Loss of N-linked glycosylation at N136
rs780324680	T>C	L168P	0.820	Loss of Helix Loss of N-linked glycosylation at N172
rs2086024485	G>A	G235R	0.825	Loss of Catalytic site at C232

### 3.3 Conservation and structural impacts

**FIGURE 2:**
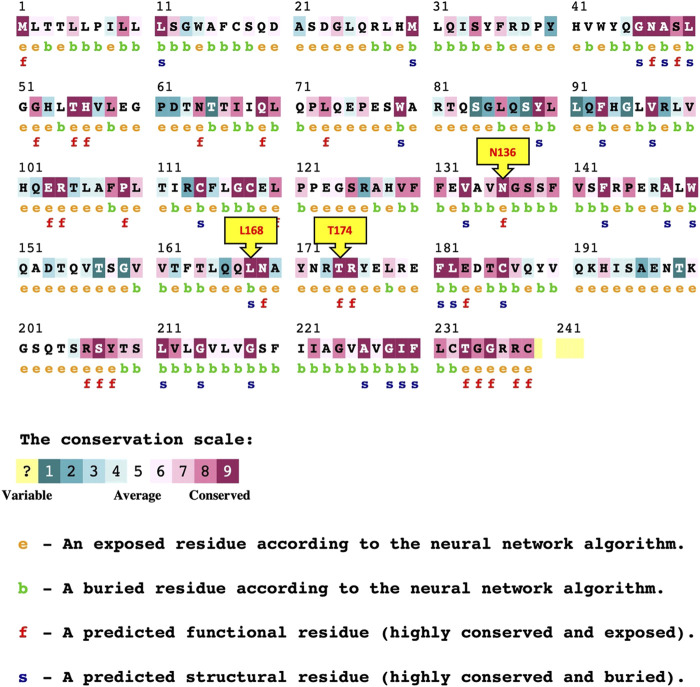
ConSurf Result for *PROCR* gene nsSNPs.

Evolutionary conservation analysis presented in (Figure 2; Table 5), using ConSurf, reveals that several mutated residues (T174I, R206P, N136I, and G235R) are highly conserved and either exposed or functional, emphasizing their critical roles in protein activity. In contrast, the L168P substitution is located in a conserved, buried region, suggesting a structural role whose alteration could have significant repercussions on the protein’s overall stability.

**TABLE 5:** Conservation Analysis of *PROCR* gene nsSNPs (ConSurf).

SNPs information			ConSurf
SNP-ID	Nucleotide Substitution	Amino Acid Substitution	Result
rs148819393	C>T	T174I	Conserved/exposed/functional
rs761318857	G>C	R206P	Conserved/exposed/functional
rs762010333	A>T	N136I	Conserved/exposed/functional
rs780324680	T>C	L168P	Conserved/Buried/structural
rs2086024485	G>A	G235R	Conserved/exposed/functional

The HOPE analysis of the structural impacts of these nsSNPs is shown in Table 6. The R206P mutation results in a decrease in residue size with an increase in hydrophobicity, leading to a loss of charge and potential disruption of key interactions. Similarly, the G235R substitution introduces an increase in size and charge with decreased hydrophobicity, likely causing steric hindrance and structural perturbations. The L168P variant is predicted to disrupt the α-helix due to its reduced size and increased hydrophobicity, while N136I may lead to destabilization of the local structure through the loss of hydrogen bonds. T174I is associated with increases in both size and hydrophobicity, which could disrupt ligand interactions and multimeric contacts. Although several mutations were predicted to have structural and functional impacts, only N136I (MAF = 0.000111/1), L168P (MAF = 0.0000038/1), and T174I (0.0000038/1) were selected to be used for molecular dynamics (MD) simulations because these mutations were located within the protein’s reactive surface area, as identified by structural visualization using PyMol. This surface is directly involved in the protein’s interaction with physiological partners, such as activated protein C. In contrast, R206P and G235R, while highly conserved and functionally important according to ConSurf and HOPE analyses, are positioned in regions distal to the active or ligand-binding interface. Their predicted effects, including steric hindrance or altered charge, are likely to affect structural integrity or folding stability rather than direct functional interaction. Therefore, we prioritized mutations that are both functionally relevant and structurally accessible to ligands for dynamic simulation.

**TABLE 6:** Structural Impacts of *PROCR* gene nsSNPs-HOPE Analysis.

SNPs information			Hope		
SNP-ID	Nucleotide Substitution	Amino Acid Substitution	Size	Hydrophobicity	Key Structural Impact
rs761318857	G>C	R206P	Decrease	Increase	Loss of charge, potential disruption of interactions due to size reduction and increased hydrophobicity
rs2086024485	G>A	G235R	Increase	Decrease	Increased size and charge introduce steric hindrance and possible structural disruption
rs780324680	T>C	L168P	Decrease	Increase	Disruption of α-helix structure, leading to potential loss of protein function
rs762010333	A>T	N136I	Decrease	Increase	Loss of hydrogen bond and destabilization of local structure
rs148819393	C>T	T174I	Increase	Increase	Potential disruption of ligand interactions and multimeric contacts due to increased size and hydrophobicity

**FIGURE 3:**
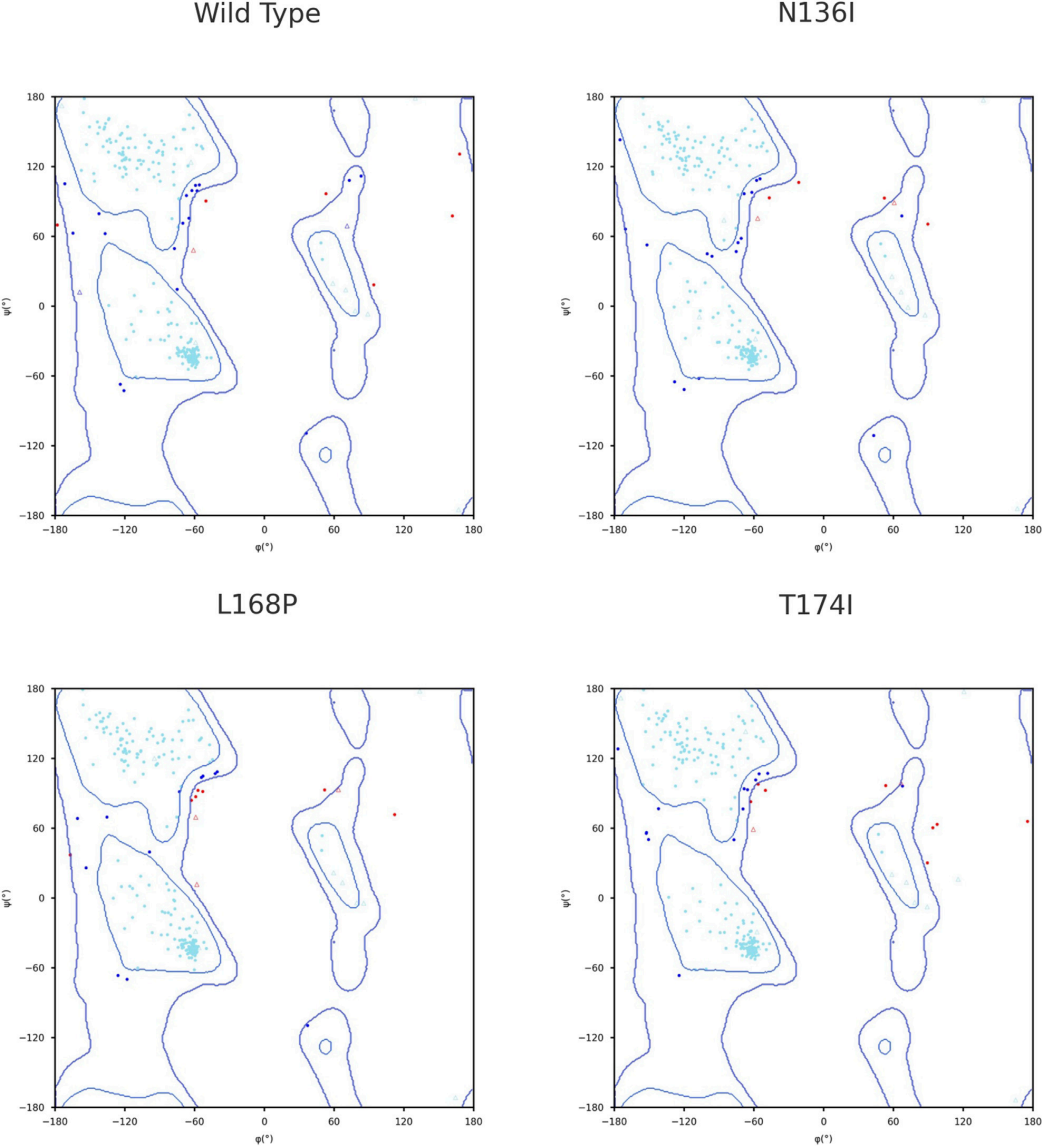
Ramachandran plot for secondary structure analysis.

To assess the conformational changes introduced by selected point mutations, Ramachandran plots were generated using STRIDE tool for the wild-type protein and the three mutant variants: N136I, L168P, and T174I. The distribution of backbone dihedral angles (ϕ, ψ) was compared to evaluate deviations in secondary structure preferences. In the wild-type structure, most residues were confined within the allowed regions corresponding to canonical α-helical and β-sheet conformations. Only a few outliers were observed, indicating a well-folded, stable backbone geometry. In the N136I mutant, the overall distribution remained comparable to the wild type; however, an increase in outliers was observed in disallowed regions, particularly near ψ ≈ 60°–90° and ϕ > 90°. This suggests a minor local disturbance in backbone flexibility, potentially due to the bulkier isoleucine side chain replacing asparagine. In the L168P mutant, a more pronounced shift in backbone angles was noted. Several residues appeared outside the favored regions, including in the disallowed upper-right quadrant. This pattern is consistent with the structural rigidity introduced by proline, which restricts ϕ angles and often disrupts α-helical segments. The data suggest that L168P induces local destabilization of secondary structure, likely disrupting helical continuity. The T174I mutant also exhibited an increased number of residues with ϕ/ψ angles in disallowed regions, though to a lesser extent than L168P. The substitution of threonine with the bulkier and more hydrophobic isoleucine may affect local folding but without significant global disruption (Figure 3).

### 3.4 Identifying protein active sites

**FIGURE 4:**
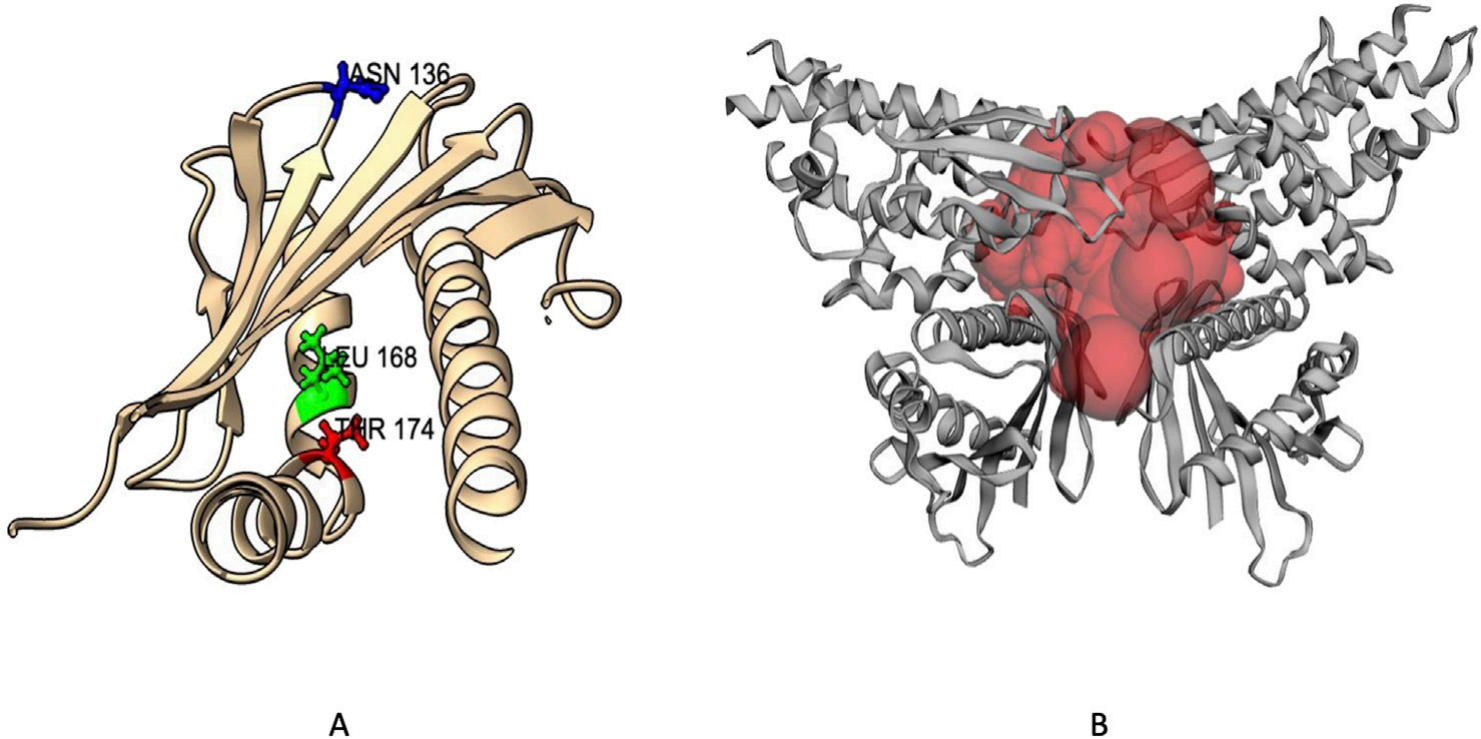
EPCR Protein Mutants within Reactive Surface: **(A)** Genetic variants highlighted in the protein 3D structure, **(B)** Multimeric protein structure showing the reactive site.

To further refine our focus, we used PyMOL to filter out mutations located outside the protein-facing portion of the reactive surface, as these are less likely to affect functionally important regions. This analysis identified three mutations—N136I, L168P, and T174I—located within the reactive surface (Figure 4). These mutations are of particular interest due to their strategic locations, which may significantly impact protein activity and stability. Focusing on these variants enhances our understanding of how specific structural changes contribute to protein dysfunction and disease pathogenesis.

### 3.5 Root mean square deviation (RMSD)

**FIGURE 5:**
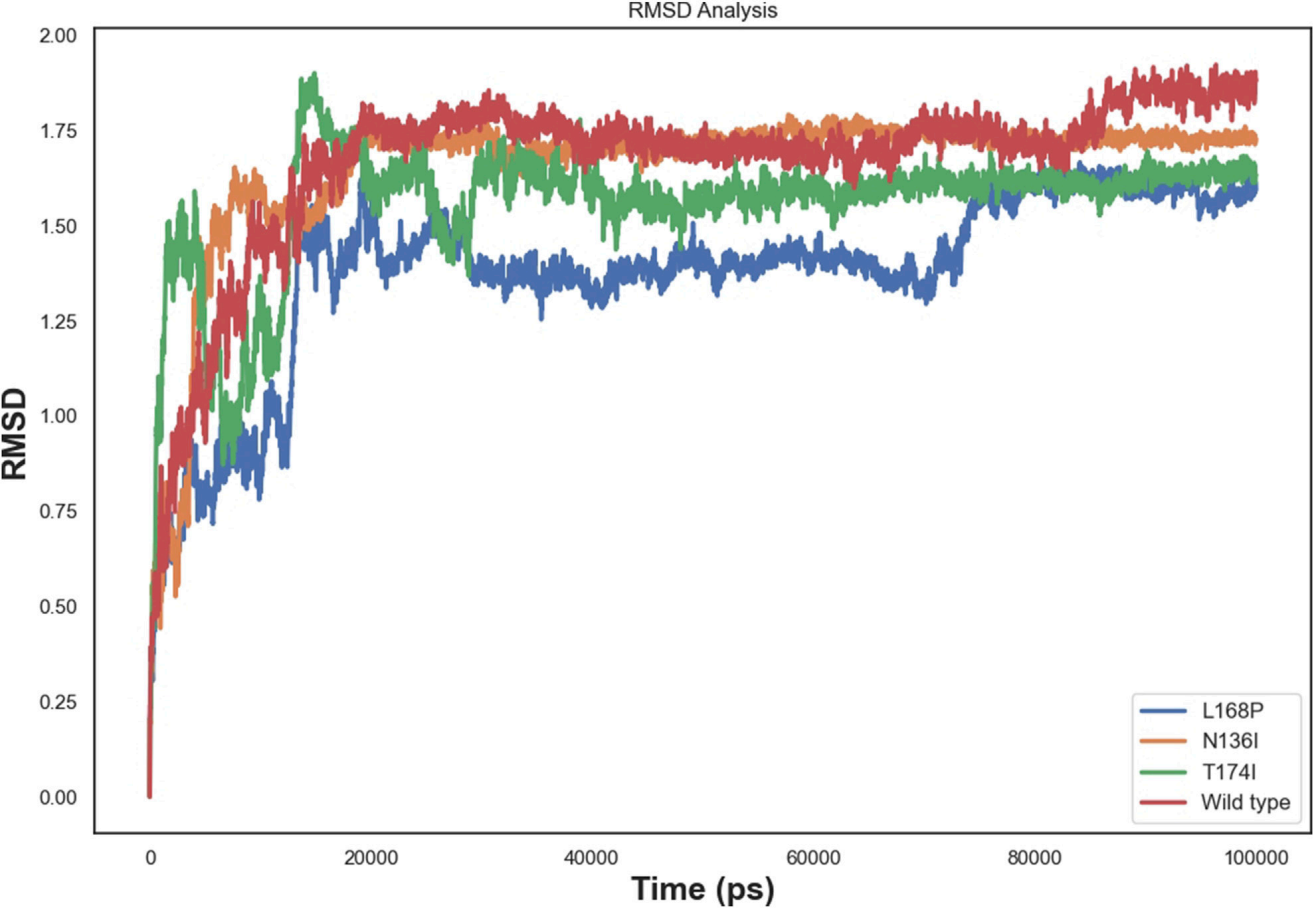
Rmsd comparison of wild-type and mutant EPCR protein.

We performed 100 ns molecular dynamics simulations to assess the effects of point mutations on protein structure, comparing the wild-type (WT) protein with three single mutants (L168P, N136I, and T174I). Analysis of the Cα RMSD (Figure 5) revealed an initial equilibration phase during the first 5–10 ns, followed by a plateau for all systems. The WT protein exhibited the highest average RMSD (∼1.7–1.8 nm), indicating greater conformational rearrangements. In contrast, L168P stabilized at a lower RMSD (∼1.5 nm), suggesting that the proline substitution confers increased rigidity and a more compact structure. Both N136I and T174I converged at intermediate RMSD values (∼1.6 nm), reflecting subtler structural changes compared to WT (Figure 5).

### 3.6 Root mean square fluctuation (RMSF)

**FIGURE 6:**
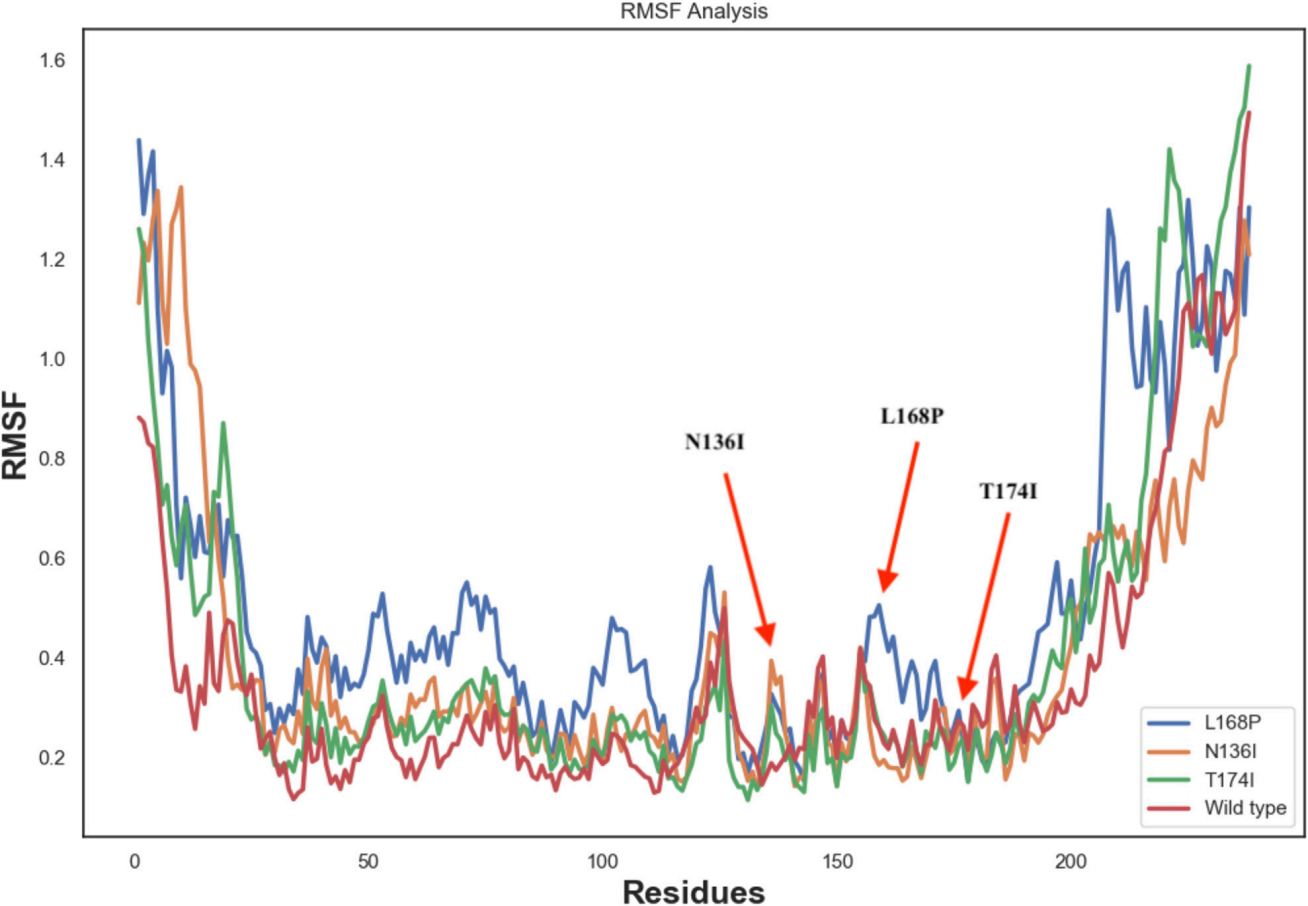
RMSF analysis of wild-type and mutant EPCR protein.

To better visualize the impact of mutations, RMSF analysis was calculated at the residue level rather than at the atom level. This approach allows clearer interpretation of local flexibility changes, particularly around the mutation sites. For instance, residue-level RMSF plots show increased fluctuations in the vicinity of residues N136, and L168, but not T174 in the mutant structures compared to WT, suggesting local destabilization. Furthermore, the differences in RMSD and SASA trends between the wild-type and mutant systems were re-examined to clarify the dynamic deviation patterns, which further corroborate the destabilizing effect of these substitutions. Correspondingly, the RMSF plots and annotated structural regions are presented in Figure 6.

### 3.7 Radius of gyration

**FIGURE 7:**
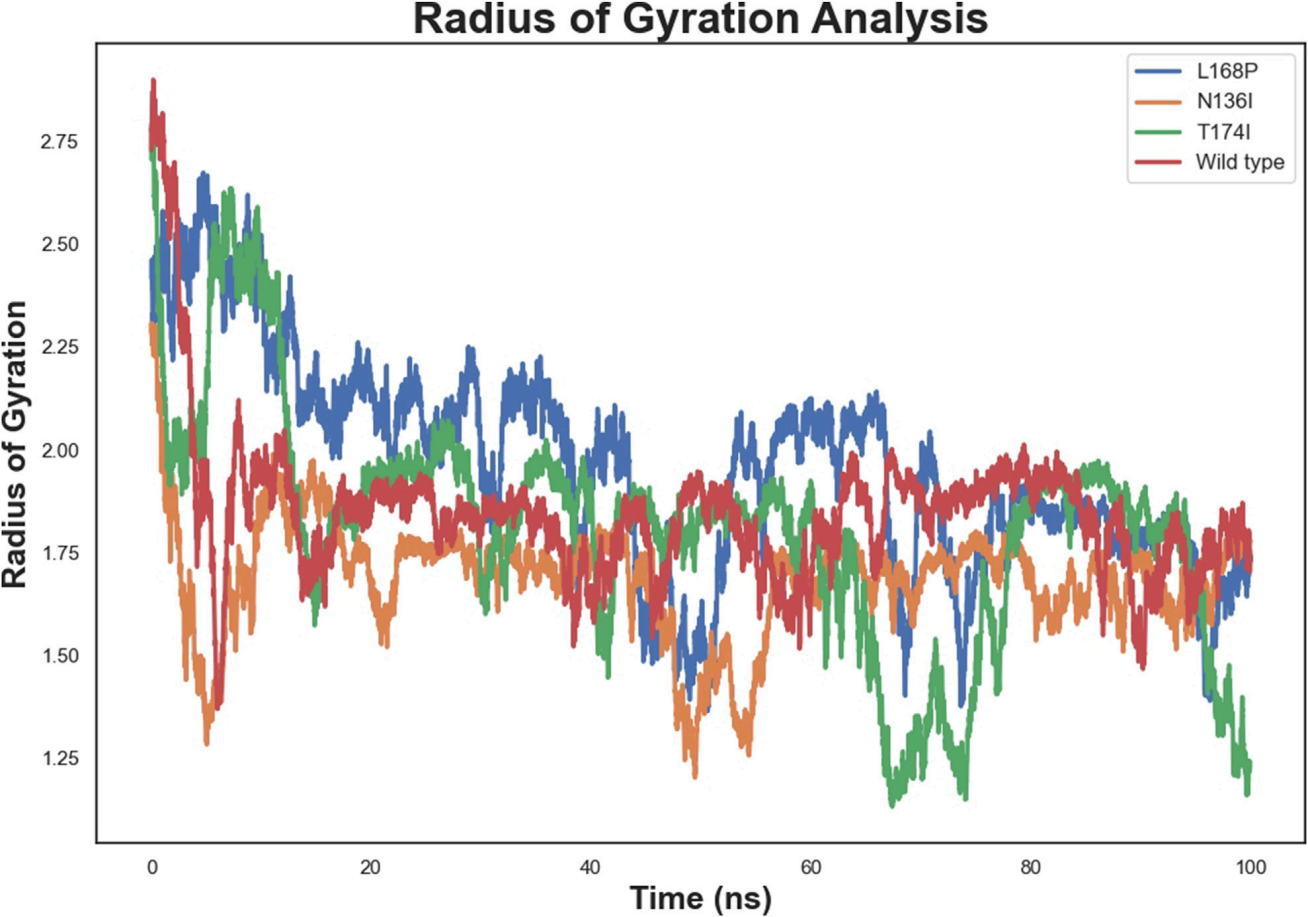
Radius of gyration of Wild-Type and Mutant EPCR Proteins.

The radius of gyration (Figure 7) further supported these findings. The WT protein stabilized at an Rg of approximately 2.2 nm, indicating moderate compactness. L168P showed a lower Rg (∼2.1 nm), and N136I converged near 2.0 nm, both suggesting increased compactness relative to WT. T174I’s Rg remained close to that of WT, consistent with minimal perturbations in global dynamics.

### 3.8 Hydrogen bond

**FIGURE 8:**
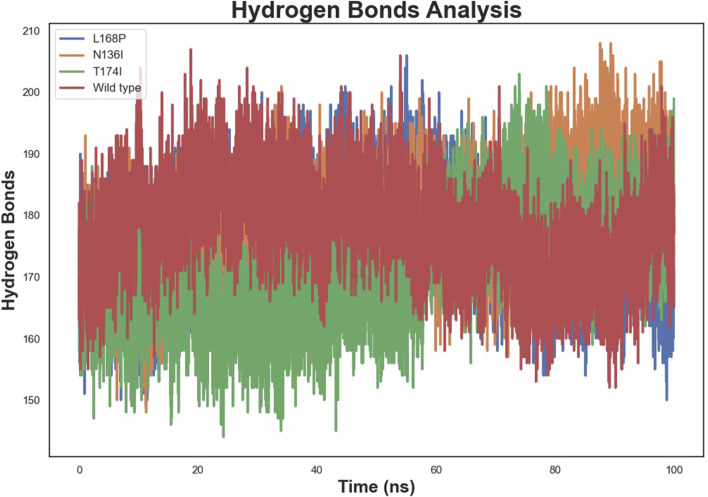
Hydrogen bond analysis of wild-type and mutant EPCR proteins.

Hydrogen bond analysis (Figure 8) revealed stable intramolecular hydrogen-bond networks post-equilibration, with average counts ranging from 150 to 200. L168P exhibited a slightly higher number of hydrogen bonds, consistent with its compact structure, while N136I showed a moderate increase relative to WT. T174I maintained a hydrogen-bond count similar to WT, suggesting that its overall hydrogen-bonding network remains largely intact despite localized fluctuations.

### 3.9 Solvent accessible surface area (SASA)

**FIGURE 9:**
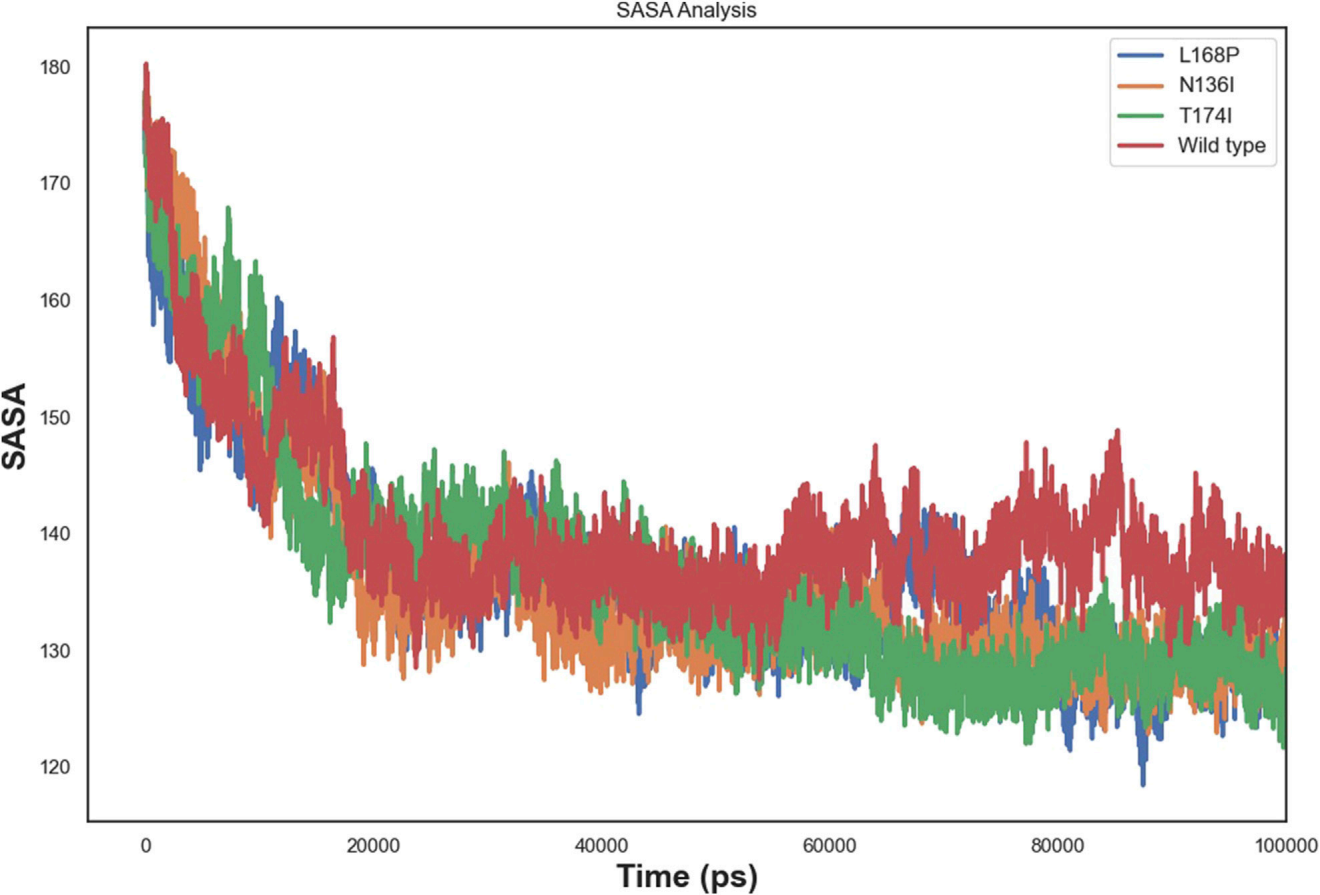
SASA analysis of wild-type and mutant EPCR proteins.

SASA analysis (Figure 9) demonstrated that all systems stabilized after equilibration. WT showed a moderate plateau, while L168P consistently recorded the lowest SASA, reflecting its compact conformation. N136I showed a slightly reduced SASA compared to WT, and T174I’s profile closely mirrored that of the WT. These results suggest that L168P significantly stabilizes the protein by promoting a more compact and rigid structure, N136I induces moderate structural compaction, and T174I exerts a minimal effect on global architecture despite localized increases in flexibility.

## 4 Discussion

Several studies have reported associations between *PROCR* gene variants and thrombotic disorders through genome-wide association studies (GWAS) and clinical case analyses. Notably, the Ser219Gly (rs867186) polymorphism has been linked to increased levels of soluble EPCR (sEPCR) and a heightened risk of venous thromboembolism (VTE) and coronary artery disease. GWAS data have also implicated *PROCR* variants in myocardial infarction, deep vein thrombosis, and pregnancy-associated thrombosis, supporting the notion that genetic alterations in EPCR can modulate thrombotic risk. These findings highlight the importance of investigating the structural and functional consequences of nsSNPs in *PROCR*, particularly those that may destabilize EPCR or impair its anticoagulant function (Medina et al., 2008; Dennis et al., 2012; Klarin et al., 2019).

Our findings indicate that deleterious nsSNPs in the *PROCR* gene significantly compromise EPCR’s structural integrity and, by extension, its anticoagulant function. The present study demonstrates that mutations such as T174I, F36C, and L168P induce distinct structural perturbations, ranging from the disruption of glycosylation sites and impairment of α-helix integrity to altered catalytic residues, that collectively may elevate thrombotic risk.

The impact of different SNPs on the secondary structure was assessed by comparing Ramachandran plots of the wild-type and variant protein structures. This approach allowed us to examine shifts in the distribution of backbone dihedral angles and determine whether the mutations affected local flexibility or imposed conformational constraints on the backbone geometry. Structural Ramachandran analysis of the *PROCR* wild-type and mutants reveals distinct backbone perturbations. The wild-type receptor shows an excellent stereochemical profile, with the vast majority of φ/ψ angles in favored α-helical or β-sheet regions and very few outliers​ (Farajzadeh-Dehkordi et al., 2023). This indicates a well-ordered native fold. The N136I mutant deviates only slightly from this norm, exhibiting a modest increase in Ramachandran-disallowed residues. Such a minor shift suggests that N136I causes only local flexibility changes without global distortion–consistent with prior observations that conservative missense changes often preserve the backbone conformation. For example, a benign p53 variant (N235S) was found to maintain an intact core and near-native φ/ψ distribution​ (Tam et al., 2020). In contrast, the L168P mutant shows pronounced Ramachandran outliers, reflecting severe backbone disruption. Proline’s cyclic structure lacks a backbone H-bond donor and restricts φ, making it a known “helix breaker” that often induces kinks​ (Yuan et al., 1994). Accordingly, L168P places several residues in disallowed quadrants, in line with reports of proline substitutions causing abrupt helical distortions​ (Yuan et al., 1994). The T174I mutation has an intermediate effect: its bulkier isoleucine side chain likely imposes steric strain, pushing nearby φ/ψ angles slightly outside preferred regions. Similar moderate deviations in Ramachandran distributions have been noted in other coagulation proteins; for instance, a single-point mutant in Factor XII showed a drop in favored-region occupancy from ∼93% to 85%, signaling a measurable conformational perturbation​ (Pathak et al., 2015). These findings underscore how each *PROCR* variant uniquely alters structural geometry–from the almost native-like backbone in N136I to the pronounced helix unwinding in L168P–mirroring trends seen in MD studies of other receptors where pathogenic mutations induce clear Ramachandran shifts while milder substitutions do not​ (Datta et al., 2015).

Molecular dynamics simulations revealed increased RMSD and RMSF values in key functional regions, indicating destabilization of the native protein conformation. Interestingly, L168P exhibited a lower RMSD than the WT, reflecting reduced conformational flexibility due to disruption of the α-helical structure. This rigidity could hinder proper ligand binding and protein-protein interactions essential for protein C activation. The T174I variant, predicted to result in the loss of N-linked glycosylation at N172, may further exacerbate destabilization by eliminating post-translational modifications critical for EPCR folding and expression. These observations are consistent with prior studies showing that subtle alterations in secondary structure and glycosylation can impact protein function and lead to disease phenotypes (Islam et al., 2019; Behairy et al., 2022).

Our study also extends previous findings by providing detailed dynamic insights that go beyond static structural predictions. The integration of multiple computational tools with molecular dynamics simulations allowed us to capture both the global and local effects of nsSNPs on EPCR. The observed decrease in intramolecular hydrogen bonds and the increase in solvent-accessible surface area in the mutant proteins underscore the potential for these variants to weaken the overall stability of EPCR. These dynamic changes suggest a mechanistic link between the molecular alterations induced by these nsSNPs and the increased thrombotic susceptibility observed in patients, a connection that has been proposed but not fully delineated in earlier reports (Roy et al., 2024; Nimir et al., 2017).

Mechanistically, our results suggest that these mutations alter the energetic landscape of EPCR, disrupting its normal equilibrium between active and inactive conformations. One possible explanation is that the loss of glycosylation and the distortion of secondary structures reduce the protein’s ability to interact with its physiological ligands, thereby impairing its role in the protein C pathway. This interpretation is supported by our conservation analysis, which shows that several of the mutated residues are highly conserved and likely critical for the protein’s functional integrity (Klarin et al., 2019). In contrast, the T174I mutation, while affecting glycosylation, appears to induce only modest global structural changes, suggesting that its impact might be more nuanced and could potentially depend on the cellular context or interactions with other molecular partners.

Structural analysis of the mutant models revealed that certain substitutions may introduce unusual kinks or distortions in the EPCR backbone that could compromise receptor function. The L168P mutation introduces a proline residue within an α-helix, which is known to act as a helix breaker due to its rigid ring structure and inability to participate in standard hydrogen bonding (Kunz et al., 2002; Li et al., 1996). This likely causes a local kink, destabilizing the helix and altering the geometry of the ligand-binding region. Similarly, the T174I mutation replaces a polar residue with a bulkier, hydrophobic isoleucine, potentially disturbing the surface topology and protein-ligand interactions. These distortions may impair the presentation of EPCR on the cell surface, alter binding affinity to activated protein C (APC) (Liaw et al., 2001), or promote enhanced receptor shedding, which has been implicated in prothrombotic conditions. Mutations that introduce kinks or destabilize secondary structure elements may also affect receptor trafficking, glycosylation patterns, or interactions with membrane microdomains, ultimately contributing to disease phenotypes such as venous thromboembolism or inflammation-related vascular damage (Ireland et al., 2005).

While our study focuses on the structural and dynamic consequences of deleterious nsSNPs in EPCR, it is important to acknowledge that differences in protein expression levels between the wild-type and variants may also significantly influence biological function. Some mutations may not primarily affect protein structure or stability but instead impact transcriptional regulation, mRNA stability, or post-translational trafficking, leading to altered cell-surface expression of EPCR. These expression differences can act as confounding factors when interpreting the functional consequences of mutations solely from a structural perspective (Gandrille, 2008). For example, even structurally stable variants could be biologically inactive if their expression is reduced or mislocalized. Therefore, our structural predictions should be interpreted in the context of these limitations and considered as complementary to, but not a substitute for, experimental validation at the transcriptional and translational levels. Future *in vitro* studies assessing expression profiles, receptor localization, and APC-binding capacity are necessary to fully understand the phenotypic impact of the predicted mutations.

It is important to note that the majority of the nsSNPs analyzed in this study are rare variants with low allele frequencies in the general population, as reported in dbSNP database. In many cases, these variants are present at a frequency of less than 0.01% and are very rarely observed in homozygous individuals. This low prevalence may limit their direct impact at the population level, and in some cases, their phenotypic consequences may only become apparent in compound heterozygous states, under specific environmental conditions, or in individuals with additional risk factors. Nonetheless, even rare variants can have high penetrance and functional significance in specific individuals or subpopulations, especially if they occur in functionally critical regions such as the ligand binding interface of EPCR. Our findings provide a framework for prioritizing such variants for further functional assays and genotype-phenotype correlation studies in thrombotic disease cohorts.

Despite the valuable insights gained, our study has limitations. Computational predictions and MD simulations offer a robust platform for prioritizing variants, but they cannot fully replicate the complexity of cellular environments. Factors such as chaperone-mediated folding, cellular localization, and *in vivo* post-translational modifications are not entirely captured. Moreover, the 100 ns simulation timeframe provides only short-term insights; extended simulations and experimental validation are needed to assess long-term effects.

Future investigations should focus on experimental validation of these computational predictions. *In vitro* expression studies, protein stability assays, and functional tests of EPCR activity in cell models would be instrumental in corroborating our findings. Moreover, expanding the simulation timescale and exploring the effects of additional interacting partners could further elucidate the clinical significance of these nsSNPs. Ultimately, these efforts may pave the way for the development of personalized therapeutic interventions targeting thrombotic disorders, as our data provide a mechanistic framework linking genetic variation in *PROCR* to altered protein function and disease risk. In conclusion, our data provide compelling evidence that deleterious nsSNPs in the *PROCR* gene destabilize EPCR, thereby impairing its anticoagulant function and increasing thrombotic susceptibility. These insights not only enhance our understanding of the molecular mechanisms underlying thrombosis but also underscore the potential of integrated *in silico* approaches to inform risk assessment and personalized treatment strategies in vascular disorders (Behairy et al., 2022; Roy et al., 2024; Nimir et al., 2017).

## 5 Conclusion

Our integrative computational analysis and molecular dynamics simulations provide strong evidence that deleterious nsSNPs in the *PROCR* gene—particularly T174I, N136I, and L168P—significantly compromise EPCR’s structural integrity and stability. These mutations disrupt key structural elements, including glycosylation sites, α-helices, and catalytic residues, leading to altered protein dynamics, reduced hydrogen bonding, and increased solvent exposure. Collectively, these perturbations likely impair EPCR’s anticoagulant function, increasing thrombotic risk. While our findings highlight the value of *in silico* approaches for prioritizing pathogenic variants and elucidating disease mechanisms, experimental validation remains essential. These insights may ultimately support improved risk assessment and the development of personalized therapies for thrombotic disorders.

## Data Availability

The original contributions presented in the study are included in the article/Supplementary Material, further inquiries can be directed to the corresponding author.
