# Supplementary material for: Impact of nonsynonymous single nucleotide polymorphisms in PROCR gene on protein stability and thrombotic risk: a molecular dynamic approach
Source: Front Genet. 2025 Apr 30;16:1580993. doi: 10.3389/fgene.2025.1580993 (PMC12075529; doi:10.3389/fgene.2025.1580993)
Supplement: Supplementary file 1 [file DataSheet1.pdf]

S1: nsSNPs of PROCR gene from NCBI database

	SNP-ID	Nucleotide Substitution	Amino acid substitution
1.	rs150846093	T>C	S88P
2.	rs867186	A>G	S219G
3.	rs61731003	G>C	V187L
4.	rs138484833	T>C	L9P
5.	rs139714129	G>T	G95C
6.	rs140612906	G>A	G117D
7.	rs141445104	G>A	R236Q
8.	rs141487483	A>G	E77G
9.	rs143131229	C>T	R236W
10.	rs144485700	C>T	P145L
11.	rs145222911	C>A<br>C>G<br>C>T	L96I<br>L96V<br>L96F
12.	rs145801152	G>A	V215I
13.	rs146420040	C>T	R113C
14.	rs148819393	C>T	T174I
15.	rs182068766	T>G	S125A
16.	rs190585174	T>C	H193Q
17.	rs199638108	C>G	F93L
18.	rs199906882	T>G	F36C
19.	rs199997508	G>A	A170T
20.	rs200235621	G>A	R173H
21.	rs200377875	G>A<br>G>C<br>G>T	R98H<br>R98P<br>R89L
22.	rs370712079	G>A	G13S
23.	rs370937355	T>A<br>T>C	F108I<br>F108L
24.	rs371977121	T>G	V42G
25.	rs372109719	C>T	T233I
26.	rs372548432	T>C	Y89H
27.	rs374766786	G>A<br>G>T	G224D<br>G224V
28.	rs375470240	A>C	E197D
29.	rs376485381	A>G<br>A>T	Q102R<br>Q102L
30.	rs536907991	A>C	N64T
31.	rs537318101	C>A	S202R
32.	rs543257656	G>A	E123K
33.	rs745873439	C>A	Q203K
34.	rs745926875	T>C<br>T>G	I229T<br>I229S
35.	rs746421195	T>C	L165P
36.	rs746777605	C>G<br>C>T	T55R<br>T55M
37.	rs746904216	C>A<br>C>T	T82K<br>T82M
38.	rs748173952	C>T	S49L
39.	rs748947928	A>G	I112V
40.	rs749468718	G>A	G46D
41.	rs749651182	A>G	N169D

42.	rs750839510	G>T	L73F
43.	rs751051139	C>A	T185N
44.	rs751558696	C>A<br>C>T	H101N<br>H101Y
45.	rs752113781	T>G	H29Q
46.	rs754071335	G>T	E75D
47.	rs754196180	G>C	V97L
48.	rs754672800	C>G	A152G
49.	rs755052209	A>G	N47S
50.	rs755138675	C>T	P76S
51.	rs755587755	T>C	L213P
52.	rs755862059	C>T	P109L
53.	rs756372458	C>G<br>C>T	H41Q<br>H41H
54.	rs757648564	A>G	M30V
55.	rs757907256	G>A	G159R
56.	rs758033850	G>C	E183Q
57.	rs758409921	T>G	W79G
58.	rs758792841	G>A	V217M
59.	rs759013787	G>A	D23N
60.	rs759357626	A>G	Y176C
61.	rs759840671	C>T	R98C
62.	rs761056226	C>A	Q74K
63.	rs761318857	G>A<br>G>C	R206H<br>R206P
64.	rs761686527	G>A<br>G>T	G13D<br>G13V
65.	rs762010333	A>T	N136I
66.	rs762351232	C>T	H94Y
67.	rs762831201	C>A<br>C>T	T204K<br>T204I
68.	rs764612882	A>G	H29R
69.	rs764944689	A>G	E177G
70.	rs766042796	C>A<br>C>G<br>C>T	D20E<br>D20E<br>D20D
71.	rs766316108	G>A<br>G>T	R175Q<br>R175L
72.	rs766660257	A>G	Q74R
73.	rs767198659	C>G<br>C>T	S210W<br>S210L
74.	rs767883054	C>T	P72S
75.	rs767986456	G>A	R37H
76.	rs768578918	T>C	I8T
77.	rs769150733	C>G<br>C>T	A196G<br>A196V
78.	rs769689686	A>C	Q203P
79.	rs769818051	T>A	L231Q
80.	rs771055696	G>A<br>G>C	S202N<br>S202T
81.	rs772304355	A>G	T199A
82.	rs772920052	G>T	A155S
83.	rs773835107	A>G	T105A
84.	rs773990421	A>C	H56P

85.	rs774060495	C>A<br>C>T	R206S<br>R206C
86.	rs774624615	A>C	E132D
87.	rs776455706	G>A	V57M
88.	rs777035768	C>T	P122L
89.	rs777663438	C>A	L54I
90.	rs778433715	G>A<br>G>T	R113H<br>R113L
91.	rs778556804	C>A<br>C>G	D153E<br>D153E
92.	rs778798174	C>A<br>C>T	A48E<br>A48V
93.	rs779115432	G>A	E77K
94.	rs779598706	G>A<br>G>C	R104Q<br>R104P
95.	rs780274382	A>C	Q45P
96.	rs780324680	T>C	L168P
97.	rs781203431	C>G	Q102E
98.	rs781289974	C>T	A226V
99.	rs781305197	A>G<br>A>T	D23G<br>D23V
100.	rs866390428	T>C	L86P
101.	rs897396920	C>A	Q166K
102.	rs900068008	C>G<br>C>T	R173G<br>R173C
103.	rs906252917	A>G	D201G
104.	rs911196277	G>A	G159E
105.	rs914304189	G>A<br>G>T	D38N<br>D38Y
106.	rs941345346	T>G	V161G
107.	rs963148263	G>A	S219N
108.	rs966013036	G>A	G52R
109.	rs986635324	A>C<br>A>G	Q19P<br>Q19R
110.	rs997802639	T>C	L58P
111.	rs1029190106	T>C	L6P
112.	rs1052528813	G>A	R144Q
113.	rs1156549697	G>A<br>G>T	G52E<br>G52V
114.	rs1158819508	A>G	N169S
115.	rs1160641612	G>T	V225L
116.	rs1169197864	G>A	A80T
117.	rs1177460495	C>G	Q167E
118.	rs1178696131	G>A	A21T
119.	rs1179590977	A>G	Y208C
120.	rs1186019882	G>C	R27T
121.	rs1192315766	C>A	Q92K
122.	rs1197509586	T>C	F181L
123.	rs1205788761	G>T	G60V
124.	rs1207321390	T>G	L90R
125.	rs1209770143	G>A	G218D
126.	rs1219291808	T>C	S158P
127.	rs1219803790	C>G	L182V
128.	rs1222262364	C>G	L90V

129.	rs1234834964	G>C	Q71H
130.	rs1238895921	C>A<br>C>T	105TN<br>T105I
131.	rs1240895486	C>T	P7L
132.	rs1256000226	C>G<br>C>T	T199R<br>T199M
133.	rs1263301794	C>T	L202F
134.	rs1265727300	G>A	R179Q
135.	rs1271360023	C>G	Q87E
136.	rs1275956595	A>G	Y89C
137.	rs1276031058	C>T	P39S
138.	rs1277077407	C>T	R144W
139.	rs1282633530	C>G	Q69E
140.	rs1287887738	C>T	P109S
141.	rs1291731173	T>C	L11P
142.	rs1293966042	T>G	I68M
143.	rs1295236165	T>C	L91P
144.	rs1302269140	G>A	S84N
145.	rs1310905610	G>A	R237Q
146.	rs1317140168	C>T	R104W
147.	rs1326532678	C>T	T4I
148.	rs1335119169	C>T	A37C
149.	rs1336590875	G>A	M30I
150.	rs1337051566	C>A	S158Y
151.	rs1338926717	A>G	I194V
152.	rs1346045901	T>C<br>T>G	I194T<br>I194S
153.	rs1348998632	C>A<br>C>T	L28I<br>L28F
154.	rs1349277067	C>T	P72L
155.	rs1350124811	C>T	P76L
156.	rs1358215891	G>C	E132Q
157.	rs1360118046	A>G	S205G
158.	rs1365465991	G>T	G85C
159.	rs1367576625	G>A	G201N
160.	rs1379957761	T>C	I222T
161.	rs1382741903	T>C	Y208H
162.	rs1388488537	T>C	C232R
163.	rs1388649131	A>C	T233P
164.	rs1400324901	A>G	I68V
165.	rs1422251391	A>T	S78C
166.	rs1426706436	C>T	A127V
167.	rs1434975195	G>C	S138T
168.	rs1447541077	A>G<br>A>T	H193R<br>H193L
169.	rs1452074807	C>T	R179W
170.	rs1460897838	T>C<br>T>G	L106S<br>L106W
171.	rs1471909491	G>A	R126K
172.	rs1474589326	C>G	S12C
173.	rs1477645004	C>G	S125C
174.	rs1483930722	C>G	S205R
175.	rs1555789780	C>T	T154I
176.	rs1568591387	C>T	R175W

177.	rs1600733286	A>C	T65P
178.	rs1600734800	A>C	T111P
179.	rs1600734869	T>G	V135G
180.	rs1600734880	A>G	N136D
181.	rs1600734886	A>G	S1138G
182.	rs1600735516	T>G	V227G
183.	rs2060380546	A>G	Y203C
184.	rs2085957651	A>T	M1L
185.	rs2085957683	T>C	M1T
186.	rs2085957821	C>T	T3I
187.	rs2085957867	A>G	T4A
188.	rs2085957947	T>C	L55S
189.	rs2085958129	C>T	P75S
190.	rs2085988545	A>G	Q26R
191.	rs2085988938	T>C	Y35H
192.	rs2085989226	C>G	P39R
193.	rs2085989307	A>G	Y40C
194.	rs2085989439	G>A	V42M
195.	rs2085989525	T>A	Y44N
196.	rs2085989872	G>C	G51R
197.	rs2085990787	C>T	T65I
198.	rs2085990908	C>A	T66K
199.	rs2085991927	G>C	E77D
200.	rs2085993159	A>G	Q92R
201.	rs2085993193	G>C	Q92H
202.	rs2085994122	A>G	H101R
203.	rs2085994728	G>C	A107P
204.	rs2086014731	G>A	C118Y
205.	rs2086015033	G>C	E123D
206.	rs2086015460	G>A	V135M
207.	rs2086015823	G>A	V141M
208.	rs2086016137	G>C	D153H
209.	rs2086016379	C>T	T157I
210.	rs2086016792	C>A	L165M
211.	rs2086018429	T>C	Y189H
212.	rs2086018511	C>T	H193Y
213.	rs2086023149	C>T	S207F
214.	rs2086023785	T>A	F220Y
215.	rs2086024040	T>C	V225A
216.	rs2086024129	G>A	V227I
217.	rs2086024485	G>A	G235R
